# GPx1 is involved in the induction of protective autophagy in pancreatic cancer cells in response to glucose deprivation

**DOI:** 10.1038/s41419-018-1244-z

**Published:** 2018-12-11

**Authors:** Qingcai Meng, Jin Xu, Chen Liang, Jiang Liu, Jie Hua, Yiyin Zhang, Quanxing Ni, Si Shi, Xianjun Yu

**Affiliations:** 1 Department of Pancreatic Surgery, Fudan University Shanghai Cancer Center, Shanghai, 200032 China; 2 Department of Oncology, Shanghai Medical College, Fudan University, Shanghai, 200032 China; 3 Shanghai Pancreatic Cancer Institute, Shanghai, 200032 China; 4 Pancreatic Cancer Institute, Fudan University, Shanghai, 200032 China

## Abstract

Given the dense stroma and poor vascularization, access to nutrients is limited in the microenvironment of pancreatic ductal adenocarcinoma (PDA). PDA cells can efficiently recycle various metabolic substrates through the activation of different rescuing pathways, including the autophagy pathway. However, the precise roles of autophagy in cancer metabolism are not yet fully understood. In the present study, we first monitored the effect of glucose deprivation on autophagy and on the expression of glutathione peroxidase-1 (GPx1) in PDA cells under the glucose-free environment. Glucose starvation induced progressive autophagy activation in PDA cells via the activation of ROS/AMPK signaling. GPx1 degradation caused by glucose deprivation led to further ROS-dependent autophagy activation. Both GPx1 overexpression and autophagy inhibition sensitized cells to starvation-induced cell death through the activation of caspase-dependent apoptosis. Moreover, GPx1 may regulate glycolysis inhibition in PDA cells under glucose-deprived conditions. In summary, this study increases our understanding of the role of GPx1 in the induction of protective autophagy in PDA cells under extreme glucose starvation and may provide new therapeutic targets or innovative therapies.

## Introduction

Pancreatic ductal adenocarcinoma (PDA) is one of the most aggressive and lethal malignancies worldwide, with a death rate nearly equal to its rate of incidence^1^. Due to the late diagnosis, high metastatic potential, and resistance to chemoradiotherapy, patients who are diagnosed with PDA have a poor prognosis, with an overall 5-year survival rate of ~6%^2^. Hence, there is a strong impetus to understand the underlying molecular mechanisms and an overwhelming need for new targets to treat this devastating disease.

As tumors increase in size, cancer cells are exposed to heterogeneous microenvironments, with some regions displaying a significant lack of critical metabolites, including oxygen, glucose, and other nutrients^3^. In particular, glucose is an indispensable nutrient under hypoxic conditions because enhanced glycolysis compensates for the lack of energy production by aerobic metabolism^4^. Glucose-deprived conditions, rather than hypoxic conditions, may be a pivotal contributing factor for the death of cancer cells in the tumor microenvironment^5^. In PDA, alterations in metabolic programs, including increased glycolysis, altered glutamine metabolism, and autophagy activation, may be particularly important for the growth and survival of cancer cells under nutrient stress conditions^6,7^. Among these pathways, glycolysis is the main metabolic pathway in the majority of PDAs^8^. Moreover, PDA cells can efficiently recycle various metabolic substrates through the activation of different salvage pathways such as autophagy and micropinocytosis^6,9^. Autophagy is an essential cellular pathway to provide intracellular energy by the degradation of unnecessary organelles and macromolecules in response to stimuli such as metabolic stress and nutrient deprivation^10,11^. Recently, there is growing evidence supporting the function of autophagy in cancer metabolism. Autophagy is normally induced by limitations in adenosine triphosphate (ATP) availability or by a lack of essential nutrients, including glucose and amino acids^12–14^. Conversely, high levels of autophagy can provide energy in some cancers even in nutrient replete conditions, and autophagy is required for cancer growth^15,16^. However, the precise roles of autophagy in cancer metabolism are not yet fully understood.

A growing amount of evidence in recent years indicates that reactive oxygen species (ROS) production and reactive nitrogen species (RNS) imbalance are induced immediately upon nutrient deprivation and represent important mediators of autophagy^17^. The regulatory pathways of autophagy in response to nutrient starvation, as well as their tight interconnection with metabolic networks and redox homeostasis, remain unclear. Glutathione peroxidase-1 (GPx1), as an antioxidant enzyme counteracting oxidative stress, has an important role in modulating intracellular ROS^18^. GPx1 has a complex effect on the development and progression of several malignancies except for PDAs^19,20^. Therefore, we asked whether GPx1 plays a role in PDAs to mediate energy stress.

As glycolysis is the main metabolic pathway in PDAs, we report in this study that extreme glucose starvation leads to progressive autophagy activation in PDA cells. The decreased GPx1 was involved in this process through the activation of ROS/AMP-dependent protein kinase (AMPK) signaling. Both GPx1 overexpression and autophagy inhibition sensitized cells to starvation-induced cell death through the activation of caspase-dependent apoptosis. Moreover, GPx1 may also inhibit glycolysis in PDA cells under glucose-deprived conditions.

## Results

### Glucose deprivation induces autophagy in PDA cells

**Fig. 1:**
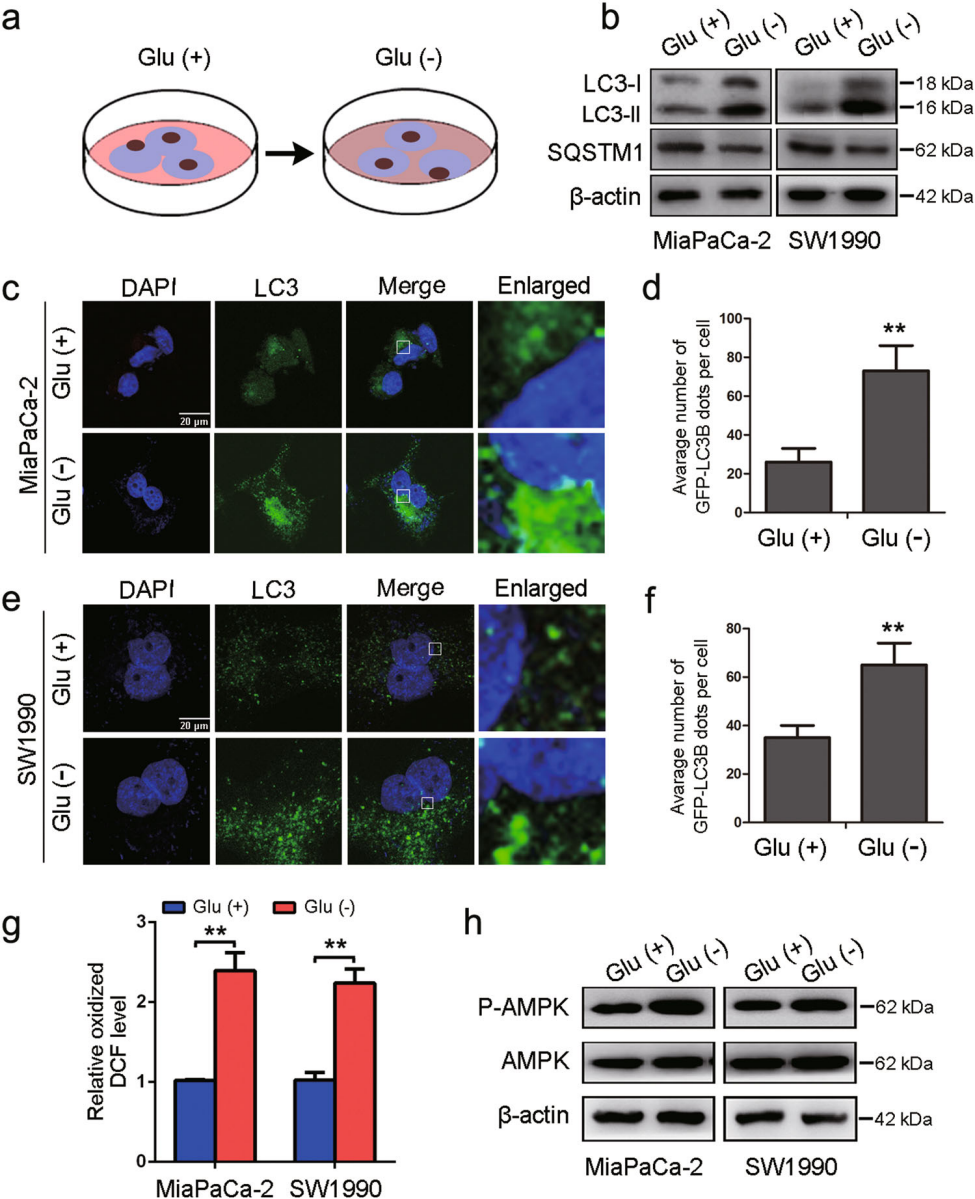
Glucose deprivation induces autophagy in PDA cells. **a** Autophagy was monitored in MiaPaCa-2 and SW1990 cells in glucose-deprived medium for 24 h. **b** MiaPaCa-2 and SW1990 cells were incubated in DMEM or glucose-free medium for 24 h. Cell lysates were immunoblotted for LC3, SQSTM1, and β-actin. **c–f** GFP-LC3-transfected PDA cells were visualized by confocal microscopy, cultured in complete or glucose-free medium for 24 h, and analyzed for LC3-expressing puncta (**P < 0.01). **g** ROS levels were measured in PDA cells in glucose-deprived medium for 24 h (**P < 0.01). **h** MiaPaCa-2 and SW1990 cells were incubated in DMEM or glucose-free medium for 24 h. Cell lysates were immunoblotted for P-AMPK, AMPK and β-actin expression

To determine the specific functional role of autophagy in cancer metabolism, we first monitored the effect of glucose deprivation on autophagy in PDA cell lines (Fig. 1a). Generally, the conversion of nonlipidated soluble LC3 (LC3-I) to phosphatidylethanolamine-conjugated LC3 (LC3-II) serves as a hallmark of autophagy^21^, and immunoblot analysis revealed a dramatic increase in the ratio of LC3-II to LC3-I in response to glucose deprivation in MiaPaCa-2 and SW1990 cells (Fig. 1b). We also performed an immunofluorescence analysis for LC3 and observed that the number of LC3-labeled vacuoles formed increased upon glucose deprivation compared with that in cells cultured under glucose-replete conditions (Fig. 1c–f). Moreover, the abundance of SQSTM1/A170/p62, an LC3-binding protein and receptor that is degraded via autophagy^22^, decreased in glucose-deprived conditions (Fig. 1b), indicating that autophagy was activated in response to glucose deprivation. Reactive oxygen species (ROS) generation is related to metabolic stress associated with nutrient deprivation and we examined and observed that the ROS levels were higher in PDA cells upon glucose deprivation (Fig. 1g). AMPK functions as a metabolic checkpoint and is typically activated by an increased AMP/ATP ratio in order to maintain energy homeostasis^23^. We demonstrated that glucose deprivation resulted in the phosphorylation of AMPK (P-AMPK) in MiaPaCa-2 and SW1990 cells (Fig. 1h).

### An indispensable role for autophagy in maintaining cell survival following glucose deprivation

**Fig. 2:**
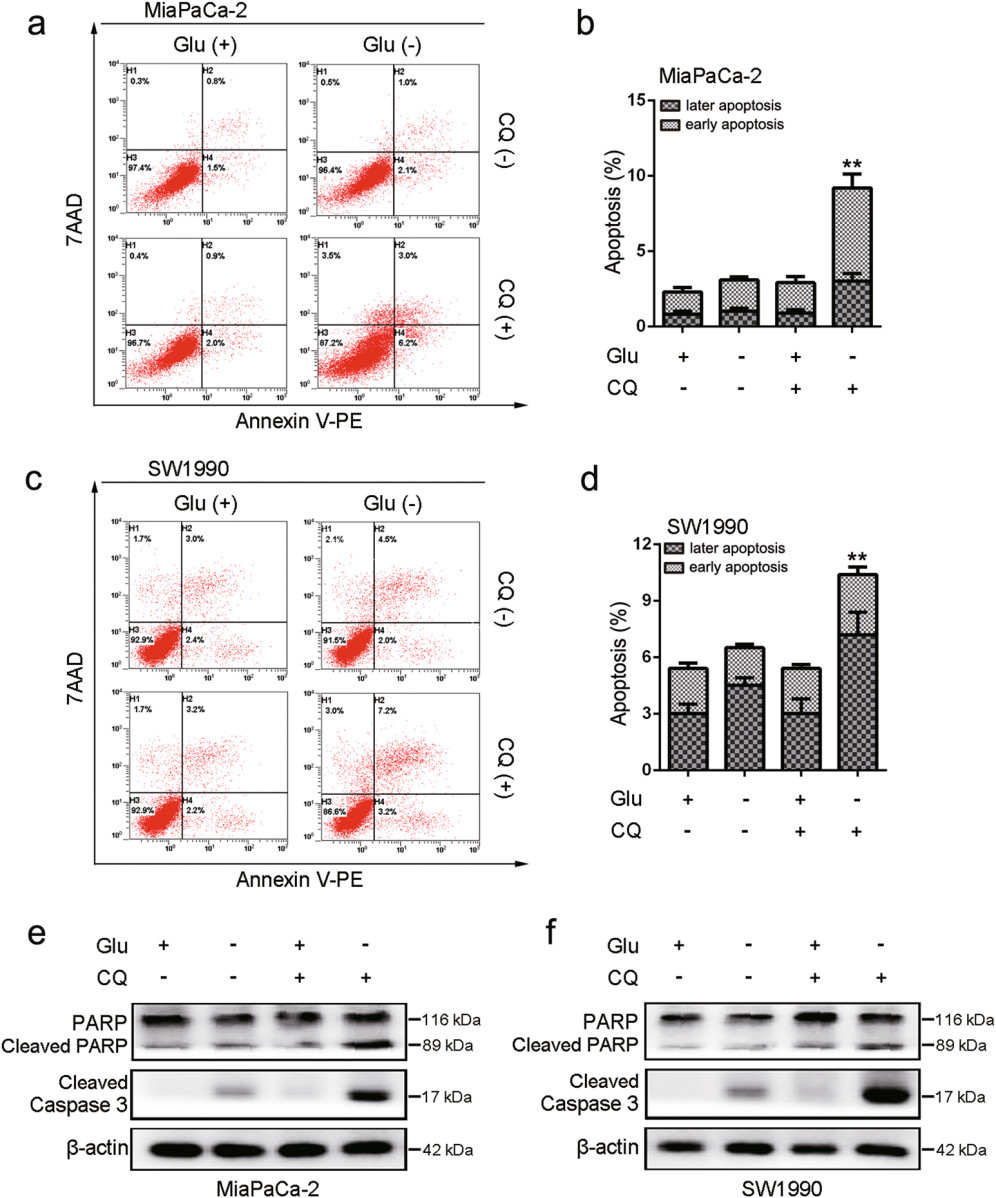
Glucose deprivation and inhibition of autophagy augments apoptotic death in PDA cells. **a**, **b** MiaPaCa-2 cells were treated with or without CQ (10 μM) in DMEM or glucose-free medium for 24 h. Apoptosis was analyzed by using the annexin V/7-AAD assay (**P < 0.01, compared with all other groups by Tukey’s post hoc test), and the same experiment was carried out in SW1990 cells (**c**, **d**). **e**, **f** MiaPaCa-2 and SW1990 cells were treated with or without CQ (10 μM) in DMEM or glucose-free medium for 24 h. Expression levels of cleaved PARP, cleaved caspase-3, and β-actin were assessed by western blotting

Because autophagy is induced in diverse stress conditions, increased autophagy might be causal or an epiphenomenon^21^. To identify the role of autophagy activation in PDA cell survival during glucose withdrawal, we treated cells with CQ (an autophagy inhibitor), which prevents fusion between autophagosomes and lysosomes. Apoptosis analysis demonstrated that either the suppression of autophagy or the deprivation of glucose had no effect on apoptotic cell death, whereas apoptotic cell death was dramatically induced upon glucose deprivation with CQ treatment (Fig. 2a–d). Consistent with these results, cleaved PARP and cleaved caspase-3 were robustly activated only when glucose was depleted by CQ treatment (Fig 2e–f). Taken together, our findings indicate that autophagy was antiapoptotic and protective against the death of PDA cells exposed to glucose deprivation.

### Glucose deprivation reduces GPx1 protein levels

**Fig. 3:**
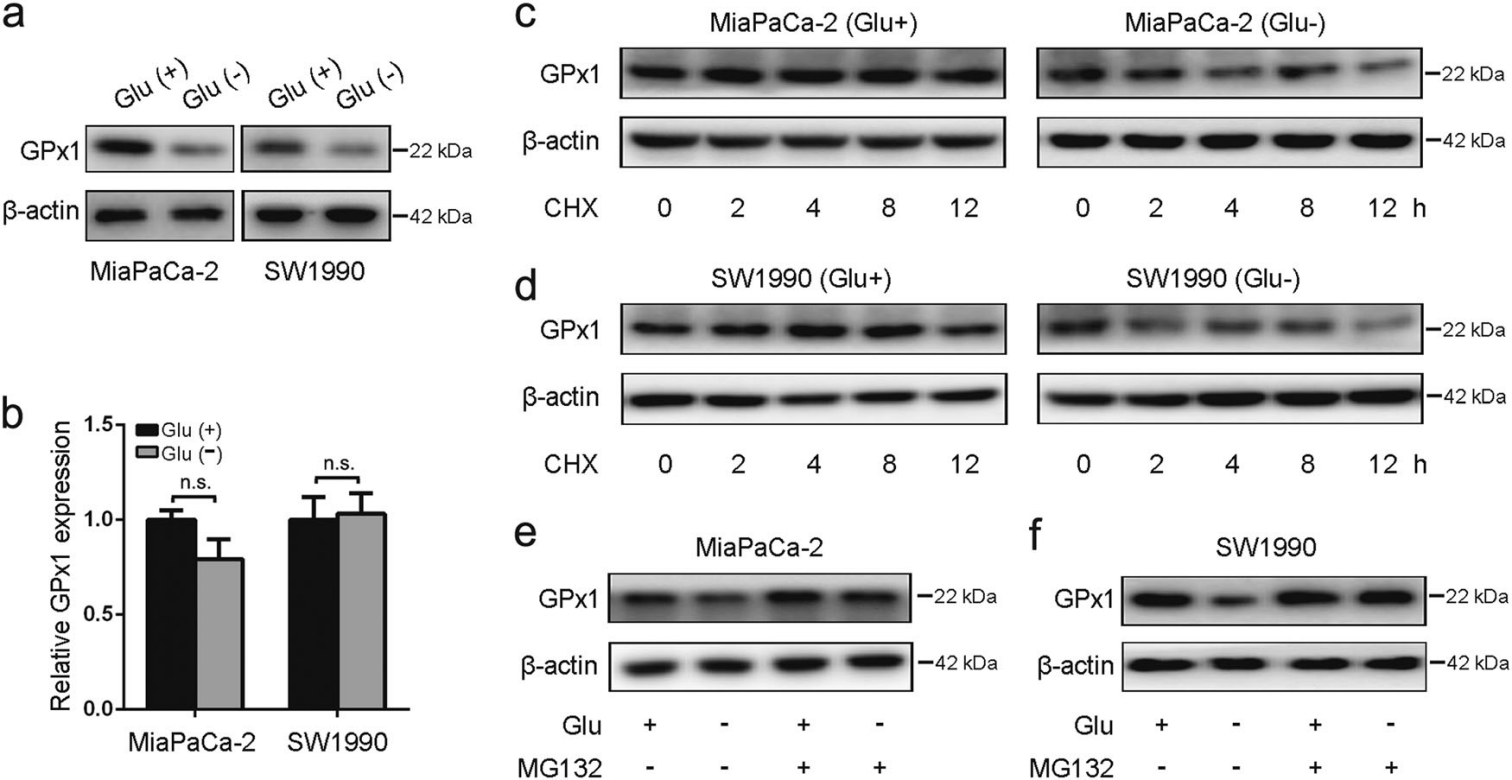
Glucose deprivation reduces GPx1 protein levels. **a**, **b** MiaPaCa-2 and SW1990 cells were incubated in DMEM or glucose-free medium for 24 h. The mRNA and protein levels of GPx1 were determined (n.s., no significance, *P < 0.05, **P < 0.01). **c**, **d** PDA cells were incubated in DMEM or glucose-free medium. Cells were treated with 100 µg/ml CHX to inhibit protein synthesis. Cells were either extracted immediately (0 h) or after 2, 4, 8 and 12 h of treatment. Cell lysates were immunoblotted for GPx1 and β-actin expression. **e**, **f** PDA cells were pretreated with 1 µmol/L proteasome inhibitor MG132 for 1 h followed by 24-h treatment with glucose-free medium or DMEM. Cell lysates were immunoblotted for GPx1 and β-actin expression

We next sought to explore whether there is a regulatory relationship between autophagy and glucose deprivation. GPx1 is an intracellular antioxidant enzyme that may modulate overall redox stress by reducing ROS, which have been proposed to serve as intracellular messengers to regulate autophagosome formation^24^. Interestingly, we observed that glucose deprivation resulted in a marked decrease in GPx1 protein levels in MiaPaCa-2 and SW1990 cells (Fig. 3a). By contrast, the mRNA levels of GPx1 were not significantly altered in PDA cells (Fig. 3b). To investigate whether glucose deprivation induces protein degradation, we analyzed the effect of cycloheximide (CHX, a protein synthesis inhibitor) on the expression of GPx1 by western blotting. PDA cells were either extracted immediately (0 h) or after 2, 4, 8, and 12 h of CHX treatment. We observed that glucose deprivation increased the degradation ratio of GPx1 (Fig. 3c, d). In addition, there was no significant decrease in GPx1 expression in PDA cells pretreated with the proteasome inhibitor MG132, which blocks the catalytic activity of the proteasome (Fig. 3e, f), suggesting that GPx1 is subject to proteasomal degradation under glucose-deprived conditions.

### Glucose deprivation leads to the decreased GPx1 protein levels, which may regulate autophagy

**Fig. 4:**
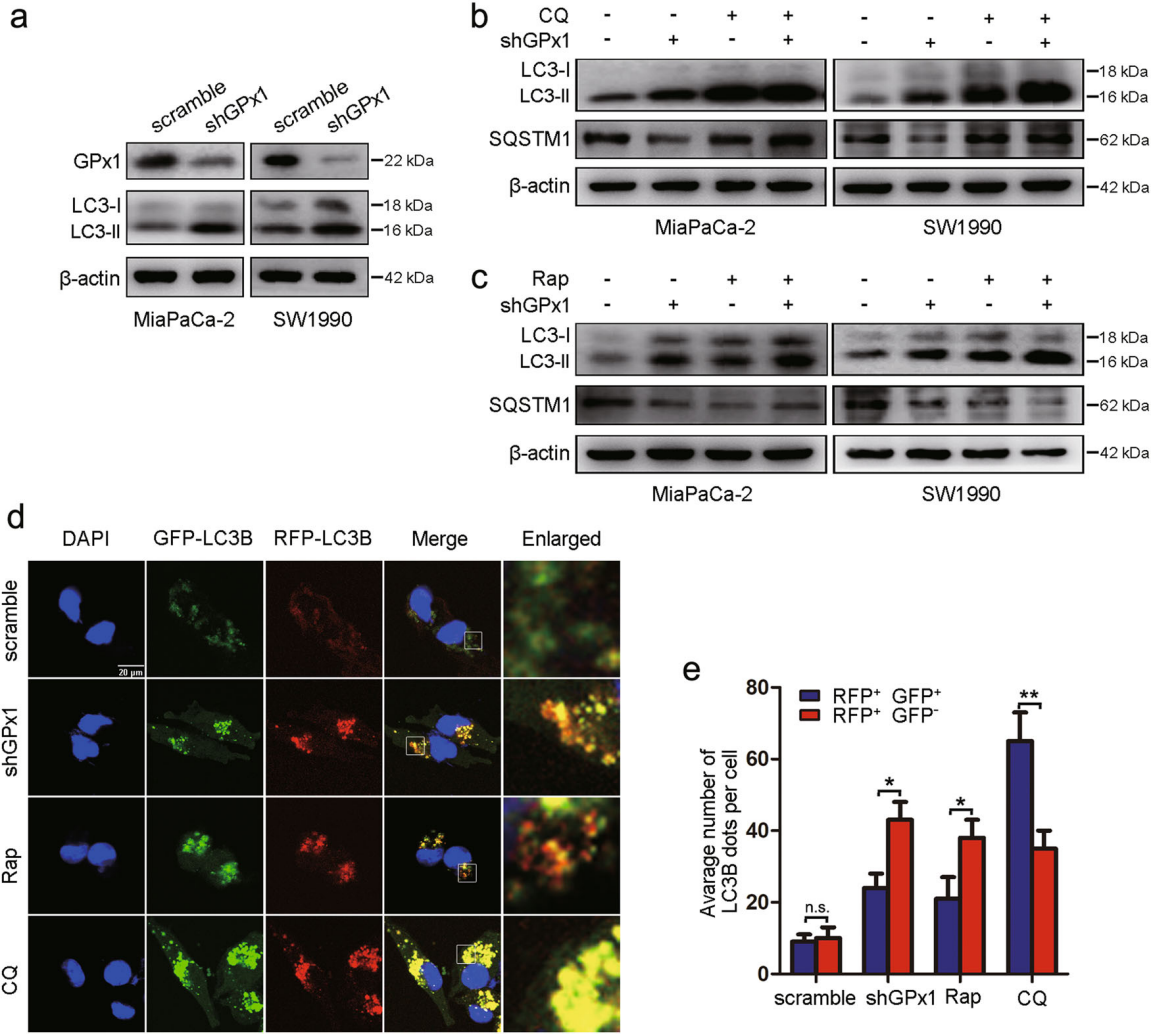
Decreased GPx1 expression induces autophagy. **a** GPx1, LC3, and β-actin expression levels were measured by immunoblot analysis in PDA cells following the silencing of GPx1 in DMEM and were compared with those in the control cells. **b** Expression levels of LC3, SQSTM1 and β-actin in GPx1-silenced cells in DMEM were examined by western blot analysis following treatment with CQ (10 μM) for 24 h. **c** GPx1-silenced cells in DMEM were treated with Rap (100 nM) for 24 h, and the indicated protein levels were then analyzed by western blotting. **d** MiaPaCa-2 cells in DMEM were transfected with GFP-mRFP-LC3B, treated with CQ (10 μM) or Rap (100 nM) for 24 h, and then observed using a confocal microscope to assess changes in green and red fluorescence (Scale bar: 20μm). The number of acidified autophagosomes (GFP^−^RFP^+^) versus the number of neutral autophagosomes (GFP^+^RFP^+^) per cell in each condition are quantified in **e** (n.s., no significance, *P < 0.05, **P < 0.01)

Thus, we hypothesized that cancer cells downregulate GPx1 to regulate autophagy in response to glucose deprivation. To confirm the role of GPx1 in this process, we detected LC3-II levels upon GPx1 knockdown in PDA cells. As expected, compared with control cells, GPx1-shRNA-expressing cells in DMEM or glucose-free medium exhibited increased levels of LC3-II protein (Fig. 4a, Supplementary Figure S1a), thus demonstrating that silencing GPx1 causes the accumulation of autophagosomes. Consistent with these findings, we also observed a negative correlation between LC3 and GPx1 levels in PDA tissues (Supplementary Figure S2a-b). Our findings regarding the clearance of SQSTM1 were consistent with these results (Fig. 4b). Autophagosome accumulation in cells, as an intermediate process within the process of autophagic flux, reflects the balance of the rates of autophagosome formation and degradation. To determine whether silencing GPx1 induces autophagic flux, cells were treated with CQ. Treatment with CQ caused significant increases in LC3-II accumulation in GPx1-shRNA-expressing cells compared with control cells in DMEM or glucose-free medium, indicating that GPx1 knockdown enhances autophagic flux (Fig. 4b, Supplementary Figure S1b). Next, treatment with rapamycin (Rap), which induces autophagy by inhibiting mTOR, also resulted in a significant increase in LC3B-II levels in GPx1-shRNA-expressing cells in DMEM or glucose-free medium (Fig. 4c, Supplementary Figure S1c). Conversely, SQSTM1 levels were markedly decreased in GPx1-shRNA-expressing cells by exposure to Rap. In addition, a tandem-labeled GFP-mRFP-LC3B construct was used to monitor autophagic flux. GFP fluorescence was quenched in autolysosomes by the low-pH environment, while RFP fluorescence was detectable in both autophagosomes and autolysosomes. The fusion of autophagosomes with lysosomes results in the loss of yellow puncta and the appearance of red-only puncta^25^. As shown in Fig. 4d, e and Supplementary Figure S1d-e, CQ inhibited the maturation of autophagy, resulting in a predominance of autophagosomes (yellow) in cells, whereas in Rap-treated or GPx1-shRNA-expressing cells, only some of the LC3-positive puncta were yellow. Taken together, these results support the idea that the levels of autophagy were further activated in the presence of glucose-free conditions and GPx1-shRNA, which indicates that the decreased GPx1 expression induced autophagic flux in PDA cells in response to glucose deprivation.

### GPx1 induces ROS-dependent autophagy activation under glucose deprivation conditions by affecting the redox state

**Fig. 5:**
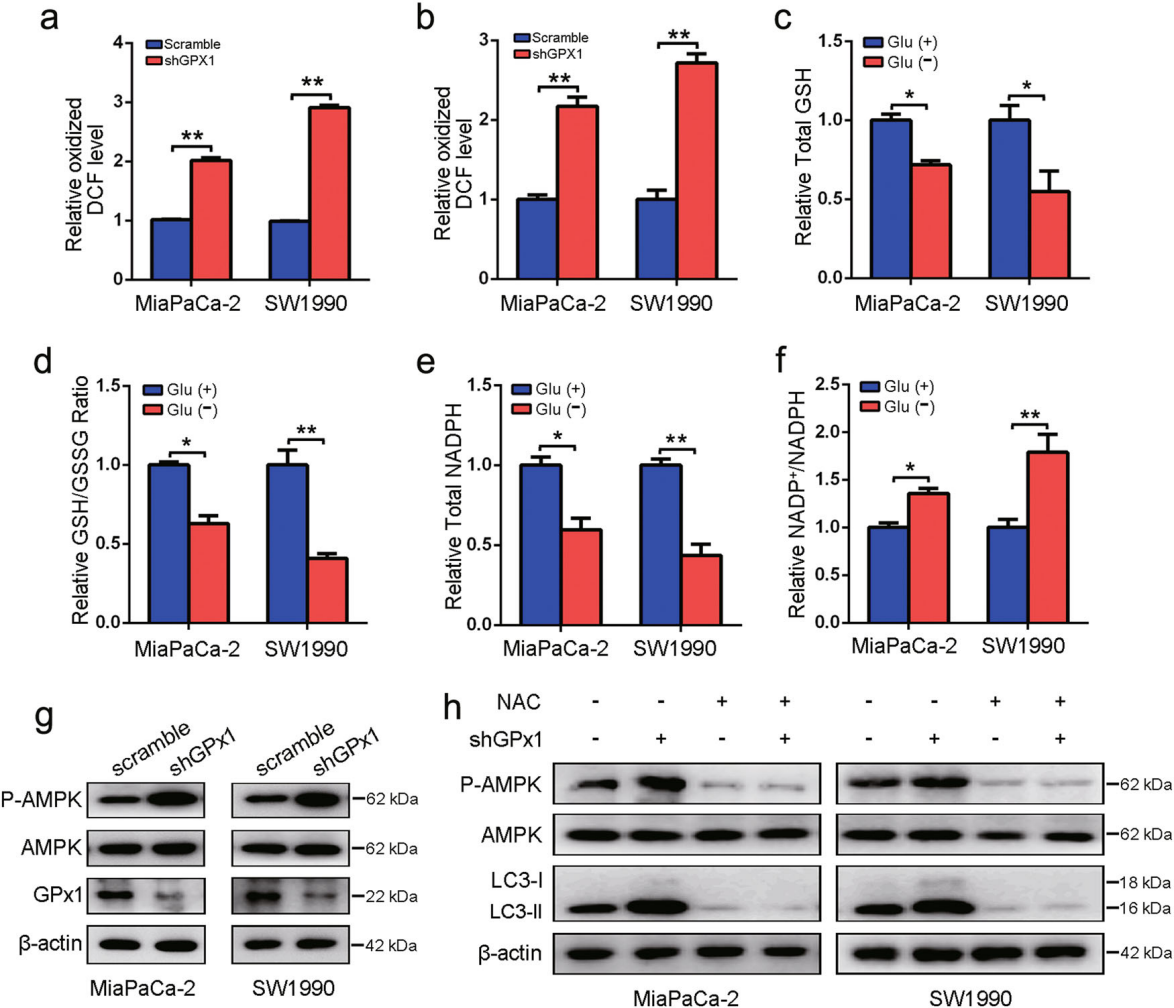
Glucose deprivation affects the redox state and autophagy through positive feedback regulation of GPx1/ROS. **a**, **b** ROS and H_2_O_2_ levels were determined in MiaPaCa-2 and SW1990 cells with GPx1 knockdown (*P < 0.05, **P < 0.01). **c**, **d** Cells were incubated in DMEM or glucose-free medium for 24 h. The total GSH level and GSH/GSSG ratio were determined (*P < 0.05, **P < 0.01). **e**, **f** Cells were incubated in DMEM or glucose-free medium for 24 h. The total NADPH levels and the NADP^+^/NADPH ratio were measured (*P < 0.05, **P < 0.01). **g** The protein levels of P-AMPK, AMPK, GPx1 and β-actin were measured by immunoblot analysis in GPx1-silenced cells and compared with those in control cells. **h** GPx1-silenced MiaPaCa-2 and SW1990 cells were treated with 20 mmol/L NAC for 1 h, and the indicated protein levels were then analyzed by western blotting

To explore the effect of GPx1 on the redox state, we first examined possible changes in ROS and H_2_O_2_ generation and observed that the ROS and H_2_O_2_ levels were higher in GPx1-knockdown PDA cells than in control cells (Fig. 5a, b). Based on the importance of GSH levels for maintaining the cellular redox balance, we evaluated the effects of glucose deprivation on the GSH/GSSG ratio. The total GSH level and GSH/GSSG ratio were significantly lower in PDA cells under glucose-free conditions (Fig. 5c, d). The low levels of GSH in the glucose-deprived environment limited the detoxification activity of GPx1 to eliminate ROS, and the degradation of GPx1 led to further ROS elevation. Next, we conducted NADP^+^/NADPH assays and demonstrated that NADPH levels were significantly decreased under glucose deprivation, whereas the NADP^+^/NADPH ratio was significantly increased by the same treatments (Fig. 5e, f). In addition, phosphorylation of AMPK was observed in GPx1-shRNA-expressing cells (Fig. 5g), suggesting that the downregulation of GPx1 resulted in the stimulation of AMPK activity by further enhancing ROS levels (especially H_2_O_2_), thus leading to autophagy. This logical speculation is supported by our findings that the antioxidant NAC abolished the GPx1-induced increases in the AMPK phosphorylation level and LC3-II expression (Fig. 5h). Taken together, the lower GPx1 protein levels caused by the increase in ROS under glucose-deprived conditions further increased ROS levels, thereby enhancing autophagy activation.

### Overexpression of GPx1 in PDA cells in glucose-free medium promotes cell death

**Fig. 6:**
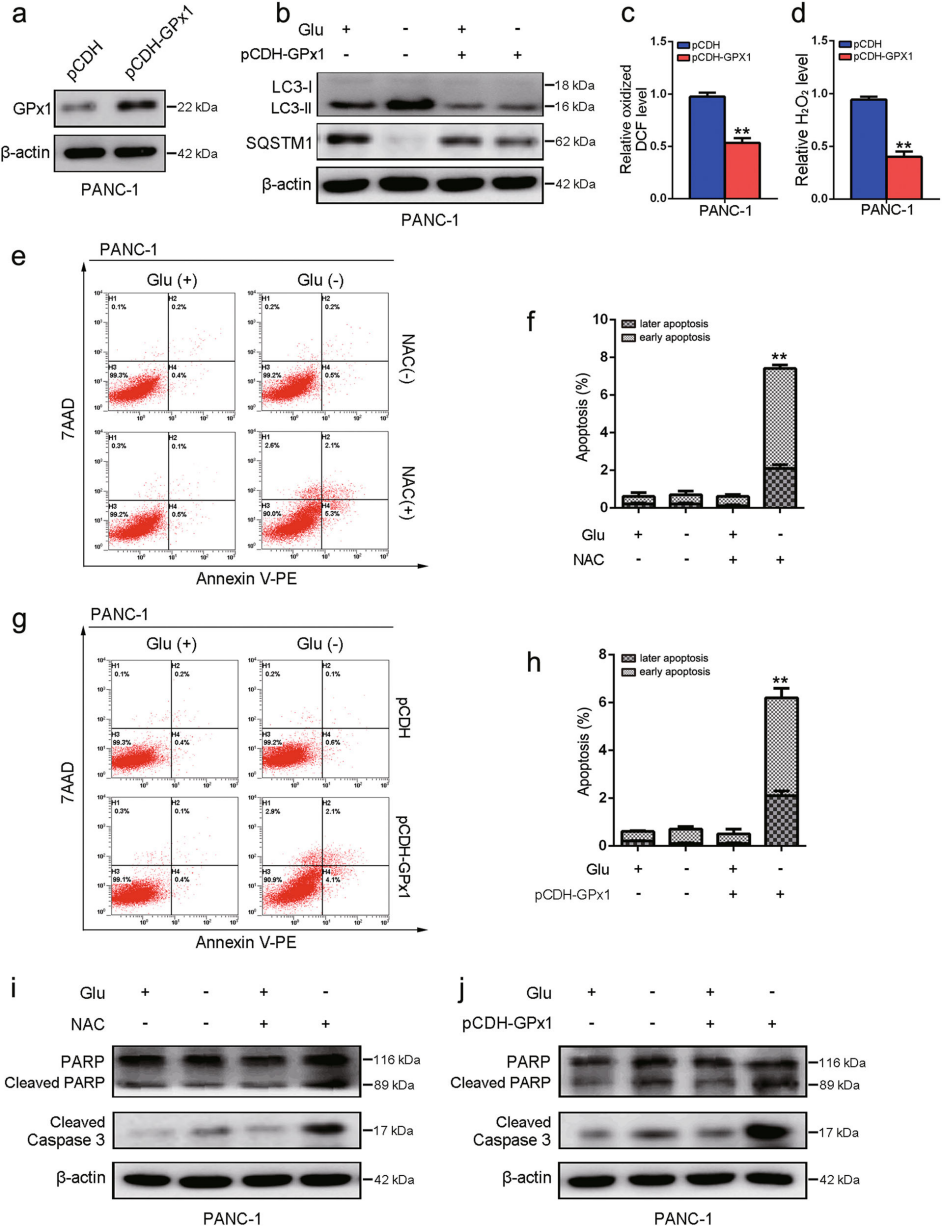
Overexpression of GPx1 in PDA cells in glucose-free medium promotes cell death. **a** PANC-1 cells ectopically overexpressing GPx1 were established. **b** The expression levels of LC3, SQSTM1 and β-actin were assessed by immunoblot analysis in PANC-1 cells with or without GPx1 overexpression in DMEM or glucose-free medium for 24 h. **c**, **d** ROS and H_2_O_2_ levels were measured in PANC-1 cells with GPx1 overexpression (**P < 0.01). **e**, **f** PANC-1 cells were treated with or without NAC (20 mM) in DMEM or glucose-free medium for 24 h. Apoptosis was analyzed by using the annexin V/7-AAD assay (**P < 0.01, compared with all other groups by Tukey’s post hoc test). **g**, **h** Apoptosis was analyzed in PANC-1 cells with or without GPx1 overexpression in DMEM or glucose-free medium for 24 h (**P < 0.01). **i** PANC-1 cells were treated with or without NAC (20 mM) in DMEM or glucose-free medium for 24 h. Cell lysates were immunoblotted for cleaved PARP, cleaved caspase-3, and β-actin. **j** Expression levels of cleaved PARP, cleaved caspase-3, and β-actin were assessed by immunoblot analysis in PANC-1 cells with or without GPx1 overexpression cultured in DMEM or glucose-free medium for 24 h

To further confirm the above results, we tested whether the overexpression of GPx1 could inhibit autophagy in PDA cells upon glucose deprivation. To test this, we constructed PANC-1 cells that ectopically overexpressed GPx1 (Fig. 6a). As expected, the levels of LC3-I/II and SQSTM1 were altered in cells upon glucose deprivation, whereas the expression of these proteins was suppressed by the overexpression of GPx1 (Fig. 6b). Moreover, we observed that ROS and H_2_O_2_ levels were significantly decreased in GPx1-overexpressing cells (Fig. 6c, d). To further identify the role of GPx1 in PDA cell survival during glucose withdrawal, apoptosis analysis demonstrated that apoptotic cell death was dramatically increased under glucose deprivation conditions upon NAC treatment or overexpression of GPx1 (Fig. 6e–h). In addition, cleaved PARP and cleaved caspase-3 were upregulated under conditions of glucose deprivation in cells, GPx1 overexpression or NAC treatment (Fig. 6i–j). Taken together, GPx1 overexpression is leading to less ROS level and less autophagy induction and so inhibiting the cells to recycle compounds which could be used to generate ATP and thus increasing cell death mechanisms under glucose deprivation.

### GPx1 is also positively correlated with glucose metabolism in PDA cells

**Fig. 7:**
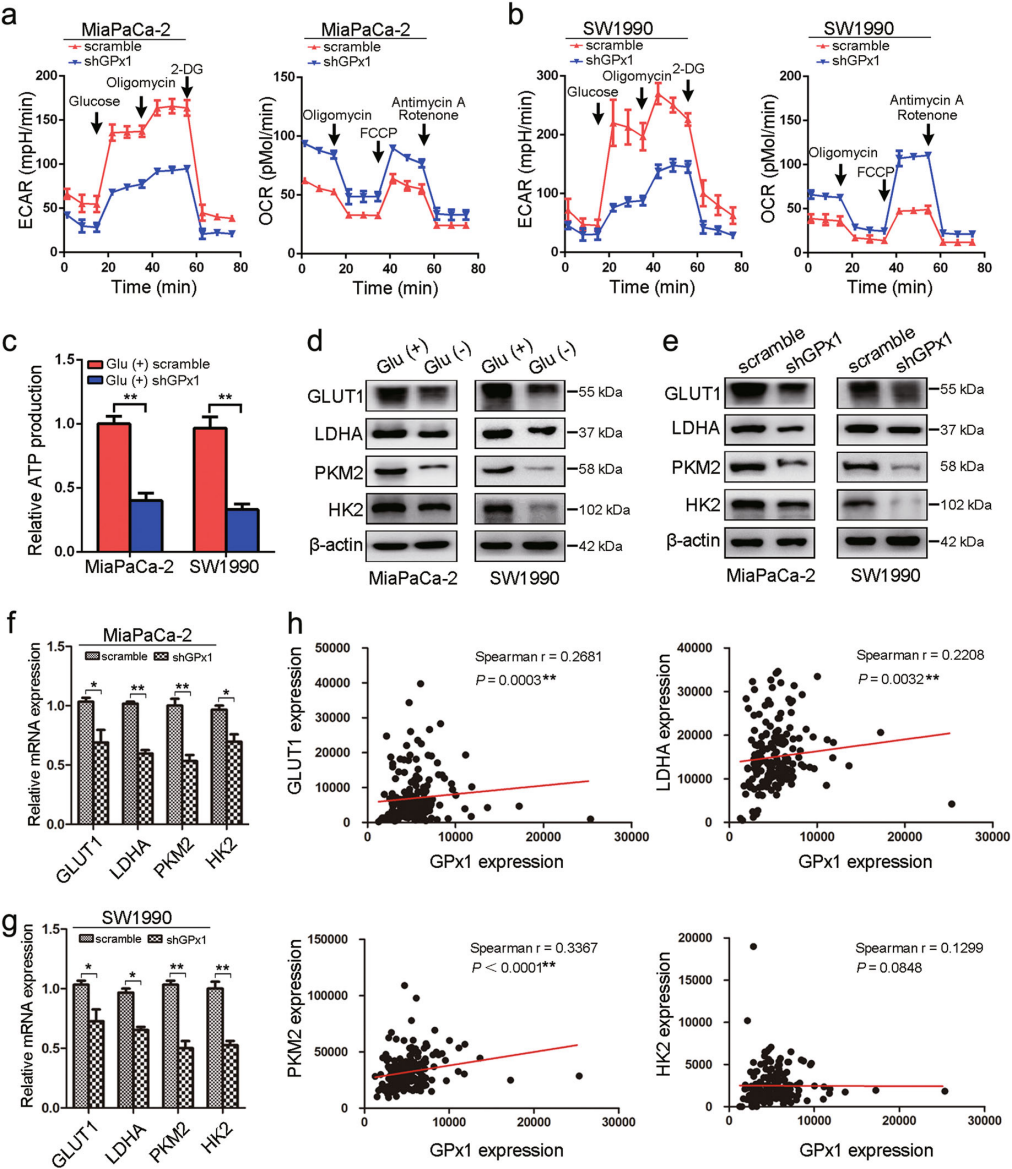
GPx1 is positively correlated with glucose metabolism in PDA cells. **a**, **b** ECAR and OCR analyses were performed in MiaPaCa-2 and SW1990 cells with GPx1 knockdown cultured in complete DMEM. **c** ATP production was measured in MiaPaCa-2 and SW1990 cells with GPx1 knockdown cultured in complete DMEM. **d** GLUT1, LDHA, PDK1, and HK2 protein levels were measured by western blotting in MiaPaCa-2 and SW1990 cells cultured in glucose-free medium. **e**–**g** The mRNA and protein levels of GLUT1, LDHA, PDK1, and HK2 were decreased by GPx1 knockdown cultured in complete DMEM (*P < 0.05, **P < 0.01). **h** Positive correlation between the expression of GPx1 and the expression of the GLUT1, LDHA, PDK1, and HK2 genes in TCGA database (**P < 0.01)

Next, to determine the impact of GPx1 expression on cellular metabolism in PDA cells upon glucose deprivation, cells were subjected to analysis with a Seahorse XF extracellular flux analyzer. In MiaPaCa-2 and SW1990 cells cultured in glucose-replete conditions, silencing GPx1 expression weakened the ECAR, while the decrease in ECAR in PANC-1 cells under glucose-free conditions was reversed by the overexpression of GPx1 (Fig. 7a, b and Supplementary Figure S3a). These results indicated that GPx1 is a positive regulator of anabolic glycolysis. In addition, cellular oxygen consumption reflects mitochondrial respiration and can be measured by the OCR. Consistent with the results of the ECAR analysis, GPx1 decreased the OCR in PDA cells, indicating that GPx1 is a negative regulator of basal mitochondrial respiration (Fig. 7a, b and Supplementary Figure S3b). Moreover, to determine the effect of GPx1 on the energy requirements for highly proliferating cancer cells under glucose starvation conditions, we then analyzed ATP production. Consistent with the above findings, ATP production was decreased in GPx1-shRNA-expressing MiaPaCa-2 and SW1990 cells, while the decrease in ATP in PANC-1 cells under glucose-free conditions was reversed by the introduction of exogenous GPx1 (Fig. 7c and Supplementary Figure S3c).

To further explore the role of GPx1 in glucose metabolism in PDA cells upon glucose deprivation, key signature enzymes in the glycolysis cascade were examined. Enzymes related to glucose transport, such as glucose transporter 1 (GLUT1), hexokinase 2 (HK2), pyruvate kinase M2 (PKM2), and lactate dehydrogenase A (LDHA), were decreased under glucose deprivation conditions (Fig. 7d). Next, the introduction of GPx1-shRNA into cells downregulated the mRNA and protein levels of these glycolytic enzymes in DMEM, indicating that GPx1 plays a positive role in anabolic glycolysis (Fig. 7e–g). The mRNA and protein levels of these glycolytic enzymes for GPx1-knockdown cells were further decreased in glucose-free media (Supplementary Figure S4a-b). Moreover, to further confirm the molecular mechanism of GPx1 in inducing alterations in glycolytic enzymes, we explored the expression data for PDA in TCGA database. Using a Spearman correlation test, we found that GPx1 expression was positively correlated with the expression of GLUT1, HK2, PKM2, and LDHA (Fig. 6h). In addition, we also determined that the expression of the enzymes GLUT1, HK2, PKM2, and LDHA decreased in tumor tissue sections from the xenografts formed by GPx1 silencing in SW1990 cells (Supplementary Figure S5). Taken together, these results suggest that GPx1 plays a vital role in glucose metabolism in PDA cells.

## Discussion

Nutrient limitation or starvation is a frequent feature of the cellular microenvironment in the core of aggressive solid tumors. Therefore, cancer cells develop rewired metabolic pathways through genetic mutations or the activation of innate recycling machinery in order to meet the increased requirements for proliferation^7,21^. In a stressed nutrient environment due to the dense stroma and hypovascularization, many PDA cells are still able to survive through adaptive alterations, including autophagy activation^16^. However, the mediators that regulate the crosstalk between autophagy and apoptotic death in cells exposed to extreme nutrient starvation are still elusive. In this study, we found that GPx1/ROS/AMPK-mediated autophagy activation rescues PDA cells in response to the energy stress of glucose-free culture, which mimics the poor tumor microenvironment. GPx1 overexpression and autophagy inhibition increased the vulnerability of PDA cells to glucose starvation-induced cell death. Furthermore, GPx1 may also inhibit glycolysis in PDA cells in glucose-deprived conditions.

Autophagy is an evolutionarily conserved cellular process that maintains energy homeostasis and mediates cellular adaptation in response to metabolic and therapeutic stresses^10,11^. In this study, we observed that autophagy was induced in PDA cells upon glucose starvation. Increasing studies have indicated that continuous autophagy provides nutrients and energy for cells through removing damaged organelles to help the survival of cancer cells in an extreme nutrient deprivation environment^26,27^. Moreover, the induction of autophagy can limit tumor growth, while the inhibition of autophagy can, at times, promote apoptotic death in cancer cells^28^. Here, our results demonstrated that an autophagy inhibitor (CQ) increased the vulnerability of PDA cells in glucose-deprived conditions to apoptotic death.

ROS, which include superoxide anion, H_2_O_2_ and hydroxyl radicals, have been copiously considered early mediators of autophagy upon nutrient deprivation^17,29^. So far, however, the specific species that induce this process remains unclear. A large body of evidence has demonstrated that H_2_O_2_, as the primary ROS, is produced immediately after starvation^30,31^. In the present study, treatment with a ROS scavenger (NAC) inhibited autophagy, indicating that ROS are crucial for autophagy execution. AMPK is the genuine sensor of the cell that responds to the energetic state, and it is directly activated by low ATP production^32,33^. To restore the energy deficiency, phosphoactivated AMPK concertedly alters catabolic pathways and stimulates autophagy by means of several distinct mechanisms^32,34,35^. Moreover, AMPK has been proposed to be activated upon H_2_O_2_ exposure, although the role of redox in the regulation of AMPK activation is still controversial^36,37^. We found that the stimulation of ROS-dependent AMPK induced autophagy in cells in response to glucose deprivation, which is consistent with the results of previous studies mentioned above.

It has been reported that glucose deficiency in can lead to an imbalance of redox homeostasis^38,39^. Indeed, we observed higher ROS levels, a lower GSH/GSSG ratio and an increased NADP^+^/NADPH ratio in PDA cells under glucose-free conditions, indicating intracellular redox imbalance. Given the role of antioxidant enzymes in this process, we also measured the protein levels of the main antioxidant enzymes upon glucose deprivation. We observed that glucose deprivation resulted in a significant decrease in GPx1 protein levels compared with superoxide dismutase (SOD1), whereas the levels of peroxisomal catalase (CAT) and paraoxonase (PON1) were not significantly altered (Supplementary Figure S6). GPx1 is a major antioxidant enzyme whose main biological role is to protect organisms from oxidative damage by scavenging hydrophilic peroxide species such as H_2_O_2_^40^. Many studies have linked GPx1 to cancer initiation and progression in various stages of carcinogenesis^41^. The reduced expression of GPx1 in lung cancer contributes to malignant transformation^42^. Previous findings indicated that GPx1 is downregulated in PDA tissues, which may be correlated with malignant biological behavior in PDA^43,44^. Interestingly, we observed that GPx1 degradation was increased in PDA cells in response to glucose deprivation. Therefore, we proposed that the decrease in GPx1 caused by glucose deprivation leads to further ROS-dependent autophagy activation.

Cancer cells are recognized to undergo specific metabolic changes to achieve optimal fitness for metabolic stress^45,46^. The Warburg effect, one of the key metabolic phenotypes, is shown by an increase in aerobic glycolysis in almost all types of tumor cells^47^. However, to date, the molecular mechanisms underlying which metabolic phenotype cancer cells choose during energy stress are not well understood. One study reported that nutrient deprivation can increase glycolysis to help cancer cells overcome metabolic stress^48^. In addition, HK2 functions as a molecular switch from glycolysis to autophagy in order to ensure cellular energy homeostasis under starvation conditions^49^. Here, glycolysis inhibition was detected in PDA cells under glucose-free conditions. Glucose metabolism is profoundly affected by oxidative stress. Excess oxidation can provoke metabolic failure, compromising cell viability by inactivating enzymes involved in glycolysis^50,51^. Therefore, we considered that the increase in ROS generated by glucose deprivation and decrease in GPx1 leads to glycolysis inhibition. Moreover, GPx1 expression was positively correlated with the expression of glycolytic enzymes in the TCGA database and our database. However, the specific regulatory mechanism requires further exploration.

**Fig. 8:**
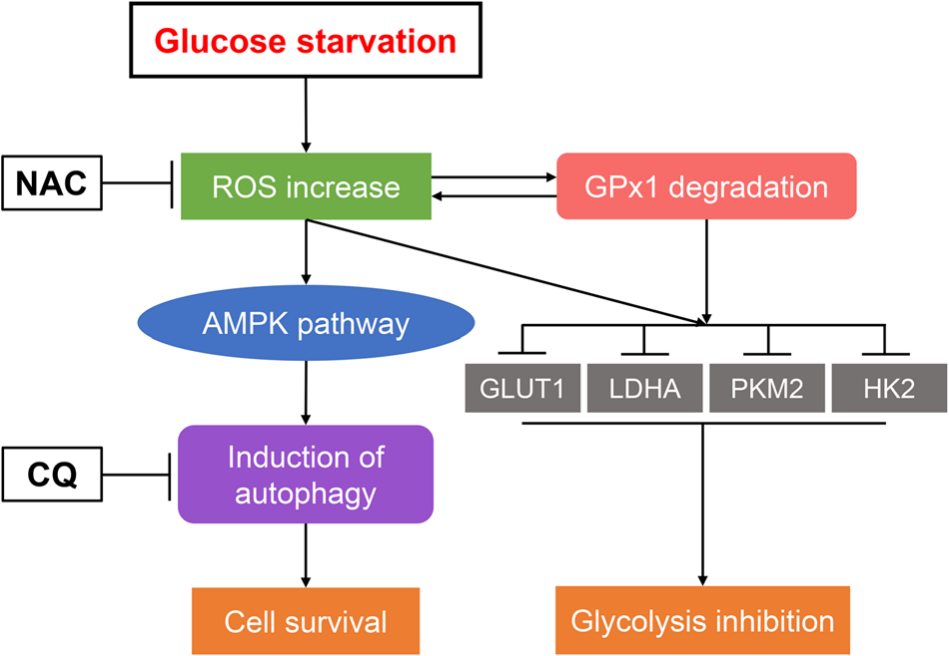
Schematic illustration summarizing the role of GPx1 in protective autophagy and glycolysis in PDA cells under glucose-free conditions

In summary, we demonstrated that decreased GPx1 is involved in the induction of protective autophagy and is regulated by glycolysis inhibition in PDA cells upon glucose deprivation (Fig. 8). These findings help us understand the role of GPx1 in the protective autophagy of PDA cells in glucose-deprived environments and may provide an appropriate therapeutic rationale for this deadly disease.

## Materials and methods

### Cell culture

The human pancreatic cancer cell lines MiaPaCa-2, SW1990, and PANC-1 were obtained from the American Type Culture Collection (ATCC) and cultured in a humidified incubator at 37 ℃ with 5% CO_2_. MiaPaCa-2 cells were cultured in Dulbecco’s Modified Eagle Medium (DMEM) supplemented with 10% fetal bovine serum (FBS) and 2.5% horse serum. SW1990 cells were cultured in L-15 medium supplemented with 10% FBS. PANC-1 cells were cultured in DMEM supplemented with 10% FBS.

### Reagents and antibodies

All commercial antibodies and chemicals were purchased from the following resources: the anti-LC3B (#3868), anti-SQSTM1/p16 (#23214), anti-cleaved PARP (#5625), and anti-cleaved caspase 3 (#9664) antibodies were from Cell Signaling Technology (CST, Danvers, MA, USA); the anti-GLUT1 (66290-1-lg), anti-HK2 (22029-1-AP), anti-LDHA (19987-1-AP), anti-PKM2 (15822-1-AP), anti-SOD1 (10269-1-AP), anti-PON1 (18155-1-AP), anti-CAT (21260-1-AP), and anti-β-actin (Proteintech, 60008-1-lg) antibodies were manufactured by Proteintech (Chicago, IL, USA); the anti-p-AMPK (ab133448), anti-AMPK (ab32047) and anti-GPx1 (ab22604) antibodies were from Abcam (Cambridge, MA, USA); Alexa Fluor 488 goat anti-rabbit IgG was purchased from Jackson ImmunoResearch; and chloroquine (CQ), rapamycin (Rap), N-acetyl-L-cysteine (NAC), cycloheximide (CHX) and MG132 were purchased from Sigma (St. Louis, MO, USA). The chemicals were dissolved in either the appropriate medium solution or dimethyl sulfoxide (DMSO) and were then prepared at the required working dilution.

### Western blot analysis

Upon treatment, cell pellets were washed twice with ice-cold PBS and lysed on ice in RIPA buffer (150 mM NaCl; 1% Triton X-100; 0.5% deoxycholate; 0.1% SDS; 50 mM Tris, pH 7.5; and protease inhibitor cocktail). The protein concentration was determined using a bicinchoninic acid protein assay kit (Beyotime, Shanghai, China). Equivalent amounts of protein (20 μg) from each sample were subjected to electrophoresis on a SDS-polyacrylamide gel and were then transferred onto polyvinylidene difluoride membranes for subsequent blotting with specific antibodies.

### Plasmid and lentivirus production

The lentiviral pLKO.1 TRC cloning vector (plasmid 10878; Addgene) was used to generate shRNA constructs targeting GPx1. Sequences twenty-one base pairs in length targeting GPx1 were GCAAGGTACTACTTATCGAGA and GCATCAGGAGAACGCCAAGAA. The pCDH-CMV-MCS-EF1-puro plasmid (SBI) was used to generate FLAG-tagged GPx1 overexpression constructs. pLKO.1 scrambled shRNA (plasmid 1864; Addgene) and the empty pCDH-CMV-MCS-EF1-puro vector were used as control vectors. The tandem-labeled GFP-mRFP-LC3B plasmid was purchased from Addgene (#21074). For transfection experiments, cells were seeded into six-well plates overnight and were transiently transfected using Lipofectamine 2000 (Invitrogen, Carlsbad, CA, USA) according to the manufacturer’s instructions. The cells were incubated with the indicated reagents for further experiments after 48 h of transfection.

### RNA isolation and quantitative real-time PCR

Total RNA was isolated by using TRIzol reagent (Invitrogen, Carlsbad, CA, USA). cDNA was obtained by using Takara’s PrimeScript RT Reagent Kit (Takara, Shanghai, China). qPCR analyses were performed using SYBR® Premix Ex Taq™ II (Takara). The expression status of the candidate genes and β-actin was determined using an ABI 7900HT Real-Time PCR System (Applied Biosystems, Inc., USA). The relative quantification value for each target gene was expressed as 2^−ΔΔCT^. β-Actin was used as an internal reference for the mRNAs. All reactions were run in triplicate. Primer sequences are listed in Supplementary Table 1.

### PDA tumor tissues and immunohistochemistry

Human PDA tumor tissues were obtained from pancreatic cancer patients who were diagnosed with PDA using histopathological tests at the Fudan University Shanghai Cancer Center (FUSCC, *n* = 40). All procedures were performed after obtaining approval from the Clinical Research Ethics Committee of FUSCC, and informed consent was gained from each patient prior to the analyses. Two independent pathologists conducted the strict pathological diagnoses and postoperative follow-ups. Animal tumor tissue sections were obtained from the nude mice (Shanghai SLAC Laboratory, Shanghai, China). These mice were randomly divided into two subgroups that received SW1990 cells in which GPx1 was stably silenced or that contained empty vector (*n* = 5/each group). Approximately 3 × 10^6^ cells were subcutaneously inoculated on left flank of the mice. At 6 weeks post implantation, the tumor specimens were surgically dissected, fixed with paraformaldehyde and then subjected to IHC staining. The protocol was approved by the Committee on the Ethics of Animal Experiments of Fudan University. IHC staining with antibodies against GPx1, LC3B, GLUT1, HK2, LDHA, and PKM2 was performed to detect protein expression levels using standard procedures. Protein expression levels were calculated by multiplying the positivity (0, <5% of the total cells; 1, 5–25%; 2, 25–50%; and 3, >50%) and intensity scores (0, no coloration; 1, pale yellow; 2, yellow; and 3, clay bank) and were classified as follows: negative (0, −); weakly positive (1–3, +); moderately positive (4–6, ++); and strongly positive (>6, +++).

### TCGA data acquisition and statistical analysis

The Cancer Genome Atlas (TCGA)-PAAD containing the RNA expression data (Level 3) of pancreatic cancer patients analyzed by RNA-seq by expectation–maximization was downloaded from the Cancer Genomics Brower of the University of California, Santa Cruz (UCSC; https://genome-cancer.ucsc.edu/). In total, 160 primary pancreatic cancer samples from patients with detailed expression data were chosen from the updated TCGA database according to the parameters mentioned above.

### Flow cytometry

Apoptosis was detected using a FITC Annexin V Apoptosis Detection Kit (BD, La Jolla, CA, USA). All the cells were collected by centrifugation, washed twice with chilled PBS, and resuspended in 100 μL of binding buffer. Following incubation with 5 μL of Annexin V-FITC and 5 μL of 7-AAD solution in the dark at room temperature for 15 min, 400 μL of binding buffer was added, and the mixture was shaken slightly. The cell apoptosis was detected by using a Flow cytometry (Beckman, Navios 2 L 8 C, USA). Data were analyzed with the FlowJo v10 software (Ashland, OR, USA).

### Confocal immunofluorescence microscopy

All cells were fixed for 15 min with 4% cold paraformaldehyde, permeabilized with 0.2% Triton X-100, and stained for immunofluorescence microscopy. The slides were incubated with blocking buffer for 30 min at 37 °C, followed by incubation overnight with anti-human LC3 antibody at 4 °C and then Alexa Fluor 488 goat anti-rabbit IgG at a 1:1000 dilution at ambient temperature for 1 h. Nuclear DNA was stained with DAPI (Sigma). Cells were examined with a Leica TCS SP5 confocal microscope equipped with ×10, ×20, ×40, and ×63 oil immersion objectives. Specimens were laser-excited sequentially at 405, 458, 476, 488, 514, 543, and 633 nm. Serial horizontal optical sections of 512 × 512 pixels with 2-times line averaging were taken at 0.4-μm intervals through the entire thickness of the cell. The average number of GFP-LC3 dots per cell was determined from 3 independent experiments. Ten random fields representing 200 cells were counted on each cover slide.

### Autophagic flux measurement

Autophagic flux was measured in cells transfected with Autophagy Tandem Sensor GFP-mRFP-LC3B. After 24 h, the fluorescence images were captured using a Leica TCS SP5 confocal microscope, and the autophagosomes (yellow dots) and autolysosomes (only red dots) were counted from 3 independent experiments.

### Determination of GSH and GSSG levels

Briefly, cells were harvested and then mixed with 30 μL of 5% metaphosphoric acid, followed by two cycles of freezing and thawing in liquid nitrogen and a 37˚C water bath. The supernatant was then harvested by centrifugation for further total GSH and GSSG determination using the GSH and GSSG assay kit (Beyotime, S0053) following the manufacturer’s instructions. The GSH/GSSG ratio were calculated using the following equation: GSH/GSSG = [Total GSH-(2 × GSSG)]/GSSG.

### Determination of the NADP^+^/NADPH ratio

Cells were harvested, and 200 μl of NADP^+^/NADPH extract was added. After mixing, cell cracking and centrifugation, the supernatant was harvested for further NADPH detection using the NADP^+^/NADPH assay kit (Beyotime, S0179) following the manufacturer’s instructions. The NADP^+^/NADPH ratio were calculated using the following equation: NADP^+^/NADPH = [Total NADPH-NADPH]/NADPH.

### Detection of ROS and H_2_O_2_ levels

We measured the ROS levels using an ROS Assay Kit (Beyotime). Cells were incubated with DCFH-DA for 1 h, and the DCF fluorescence intensities were then monitored by Flow cytometry. A Hydrogen Peroxide Assay Kit (Beyotime) was used to detect the H_2_O_2_ levels. This reagent was added to 50-μL protein samples from the prepared cells for 30 min, and the absorbance was recorded at a wavelength of 560 nm.

### ATP production analysis

The ENLITEN ATP Assay System (Promega, Madison, WI, USA) was used according to the manufacturer’s instructions. Cells were seeded into 24-well plates at an initial density of 3 × 10^4^ cells/well the day before determination. Cells were harvested by digestion with trypsin-EDTA (Gibco, Grand Island, NY, USA) and were then resuspended in PBS. ATP was extracted by adding 5% trichloroacetic acid (TCA), and the TCA was then diluted to a final concentration of 0.1% with Tris-acetate buffer (pH 7.75). The luminescence of the diluted sample (40 ml) mixed with an equal volume of rL/L reagent (Promega) was measured. The standard ATP regression curve was created using the ATP standard solution supplied in the kit. Then, the relative ATP concentration was determined and normalized to that of the control group.

### ECAR and OCR analysis

The Seahorse XF Glycolysis Stress Test Kit and Cell Mito Stress Test Kit were used to continuously monitor media acidification (ECAR) and oxygen consumption (OCR) in a Bioscience XF96 Extracellular Flux Analyzer. Cells were plated in XF96 cell culture microplates (Seahorse Bioscience) at an initial cellular density of 4 × 10^4^ cells/well the day before determination. The Seahorse buffer for cells cultured in DMEM consisted of DMEM, 25 mM glucose, phenol red, 2 mM sodium pyruvate, and 2 mM glutamine. The Seahorse buffer for cells cultured in glucose-deprived medium consisted of glucose-free DMEM, phenol red, 2 mM sodium pyruvate, and 2 mM glutamine. For ECAR measurement, 10 mM glucose, 1 mM oligomycin, and 100 mM 2-deoxyglucose (2-DG) were automatically added to measure the ECAR value. After monitoring baseline respiration, 1 mM oligomycin, 1 mM FCCP, 1 mM antimycin A and 1 mM rotenone were automatically injected into XF96 cell culture microplates to measure the OCR. The ECAR and OCR values were calculated after normalization to the cell number.

### Statistics

Data are presented as the means ± SD. All statistical analyses were performed using SPSS 19.0 software. Independent Student’s t-test (two-tailed) or one-way ANOVA and Tukey’s *post hoc* test were used to evaluate the data. For TCGA data analysis, Pearson correlation analysis was used to determine the correlation between the expression level of GPx1 and those of other genes. *P*-values less than 0.05 were considered statistically significant.

## Supplementary information

Supplementary Figure Legends

Supplementary Figure 1

Supplementary Figure 2

Supplementary Figure 3

Supplementary Figure 4

Supplementary Figure 5

Supplementary Figure 6

Supplementary Table 1

## References

[1] Siegel RL, Miller KD, Jemal A (2018). Cancer statistics, 2018. CA Cancer J. Clin..

[2] Midha S, Chawla S, Garg PK (2016). Modifiable and non-modifiable risk factors for pancreatic cancer: A review. Cancer Lett..

[3] Hockel M, Vaupel P (2001). Tumor hypoxia: definitions and current clinical, biologic, and molecular aspects. J. Natl. Cancer Inst..

[4] Hockel M, Schlenger K, Hockel S, Vaupel P (1999). Hypoxic cervical cancers with low apoptotic index are highly aggressive. Cancer Res..

[5] Cui H (2007). Enhanced expression of asparagine synthetase under glucose-deprived conditions protects pancreatic cancer cells from apoptosis induced by glucose deprivation and cisplatin. Cancer Res..

[6] Blum R, Kloog Y (2014). Metabolism addiction in pancreatic cancer. Cell death & Dis..

[7] Sousa CM, Kimmelman AC (2014). The complex landscape of pancreatic cancer metabolism. Carcinogenesis.

[8] Falasca M, Kim M, Casari I (2016). Pancreatic cancer: Current research and future directions. Biochim. Biophys. Acta.

[9] Perera RM, Bardeesy N (2015). Pancreatic Cancer Metabolism: Breaking It Down to Build It Back Up. Cancer Discov..

[10] Hale AN, Ledbetter DJ, Gawriluk TR, Rucker EB (2013). Autophagy: regulation and role in development. Autophagy.

[11] Rabinowitz JD, White E (2010). Autophagy and metabolism. Science.

[12] Egan DF (2011). Phosphorylation of ULK1 (hATG1) by AMP-activated protein kinase connects energy sensing to mitophagy. Science.

[13] Hardie DG, Ross FA, Hawley SA (2012). AMPK: a nutrient and energy sensor that maintains energy homeostasis. Nat. Rev. Mol. Cell Biol..

[14] Marino G (2014). Regulation of autophagy by cytosolic acetyl-coenzyme A. Mol. Cell.

[15] Guo JY (2011). Activated Ras requires autophagy to maintain oxidative metabolism and tumorigenesis. Genes & Dev..

[16] Yang S (2011). Pancreatic cancers require autophagy for tumor growth. Genes & Dev..

[17] Filomeni G, De Zio D, Cecconi F (2015). Oxidative stress and autophagy: the clash between damage and metabolic needs. Cell Death Differ..

[18] Rotruck JT (1973). Selenium: biochemical role as a component of glutathione peroxidase. Science.

[19] Arsova-Sarafinovska Z (2009). Glutathione peroxidase 1 (GPX1) genetic polymorphism, erythrocyte GPX activity, and prostate cancer risk. Int. Urol. Nephrol..

[20] Ichimura Y (2004). Increased risk of bladder cancer associated with a glutathione peroxidase 1 codon 198 variant. J. Urol..

[21] Ravikumar B (2010). Regulation of mammalian autophagy in physiology and pathophysiology. Physiol. Rev..

[22] Pankiv S (2007). p62/SQSTM1 binds directly to Atg8/LC3 to facilitate degradation of ubiquitinated protein aggregates by autophagy. J. Biol. Chem..

[23] Shackelford DB, Shaw RJ (2009). The LKB1-AMPK pathway: metabolism and growth control in tumour suppression. Nat. Rev. Cancer.

[24] Kalyanaraman B (2018). Teaching the basics of reactive oxygen species and their relevance to cancer biology: Mitochondrial reactive oxygen species detection, redox signaling, and targeted therapies. Redox Biol..

[25] Klionsky DJ (2012). Guidelines for the use and interpretation of assays for monitoring autophagy. Autophagy.

[26] Meijer AJ, Codogno P (2004). Regulation and role of autophagy in mammalian cells. Int. J. Biochem. Cell. Biol..

[27] Shen S (2011). Association and dissociation of autophagy, apoptosis and necrosis by systematic chemical study. Oncogene.

[28] Chaabane W (2013). Autophagy, apoptosis, mitoptosis and necrosis: interdependence between those pathways and effects on cancer. Arch. Immunol. Ther. Exp. (Warsz.).

[29] Filomeni G, Desideri E, Cardaci S, Rotilio G, Ciriolo MR (2010). Under the ROS…thiol network is the principal suspect for autophagy commitment. Autophagy.

[30] Scherz-Shouval R, Shvets E, Elazar Z (2007). Oxidation as a post-translational modification that regulates autophagy. Autophagy.

[31] Zhang C (2013). Calyxin Y induces hydrogen peroxide-dependent autophagy and apoptosis via JNK activation in human non-small cell lung cancer NCI-H460 cells. Cancer Lett..

[32] Hardie DG (2011). AMP-activated protein kinase: an energy sensor that regulates all aspects of cell function. Genes & Dev..

[33] Oakhill JS (2011). AMPK is a direct adenylate charge-regulated protein kinase. Science.

[34] Gwinn DM (2008). AMPK phosphorylation of raptor mediates a metabolic checkpoint. Mol. Cell.

[35] Inoki K (2006). TSC2 integrates Wnt and energy signals via a coordinated phosphorylation by AMPK and GSK3 to regulate cell growth. Cell.

[36] Desideri E, Filomeni G, Ciriolo MR (2012). Glutathione participates in the modulation of starvation-induced autophagy in carcinoma cells. Autophagy.

[37] Zmijewski JW (2010). Exposure to hydrogen peroxide induces oxidation and activation of AMP-activated protein kinase. J. Biol. Chem..

[38] Roberts DJ, Tan-Sah VP, Ding EY, Smith JM, Miyamoto S (2014). Hexokinase-II positively regulates glucose starvation-induced autophagy through TORC1 inhibition. Mol. Cell.

[39] Sun L, Shukair S, Naik TJ, Moazed F, Ardehali H (2008). Glucose phosphorylation and mitochondrial binding are required for the protective effects of hexokinases I and II. Mol. Cell. Biol..

[40] Brigelius-Flohe R (1999). Tissue-specific functions of individual glutathione peroxidases. Free Radic. Biol. & Med..

[41] Lubos E, Loscalzo J, Handy DE (2011). Glutathione peroxidase-1 in health and disease: from molecular mechanisms to therapeutic opportunities. Antioxid. Redox Signal..

[42] Ratnasinghe D (2000). Glutathione peroxidase codon 198 polymorphism variant increases lung cancer risk. Cancer Res..

[43] Cullen JJ, Mitros FA, Oberley LW (2003). Expression of antioxidant enzymes in diseases of the human pancreas: another link between chronic pancreatitis and pancreatic cancer. Pancreas.

[44] Kodydkova J (2013). Antioxidant status and oxidative stress markers in pancreatic cancer and chronic pancreatitis. Pancreas.

[45] Hirayama A (2009). Quantitative metabolome profiling of colon and stomach cancer microenvironment by capillary electrophoresis time-of-flight mass spectrometry. Cancer Res..

[46] Lin L (2015). MACC1 supports human gastric cancer growth under metabolic stress by enhancing the Warburg effect. Oncogene.

[47] Kim JW, Dang CV (2006). Cancer’s molecular sweet tooth and the Warburg effect. Cancer Res..

[48] Wu CA, Chao Y, Shiah SG, Lin WW (2013). Nutrient deprivation induces the Warburg effect through ROS/AMPK-dependent activation of pyruvate dehydrogenase kinase. Biochim. Biophys. Acta.

[49] Tan VP, Miyamoto S (2015). HK2/hexokinase-II integrates glycolysis and autophagy to confer cellular protection. Autophagy.

[50] Avery SV (2011). Molecular targets of oxidative stress. Biochem. J..

[51] Cecarini V (2007). Protein oxidation and cellular homeostasis: Emphasis on metabolism. Biochim. Biophys. Acta.

