# Supplementary material for: GPx1 is involved in the induction of protective autophagy in pancreatic cancer cells in response to glucose deprivation
Source: Cell Death Dis. 2018 Dec 11;9(12):1187. doi: 10.1038/s41419-018-1244-z (PMC6290009; doi:10.1038/s41419-018-1244-z)
Supplement: Supplementary file 1 — Supplementary Figure Legends [file 41419_2018_1244_MOESM1_ESM.docx]

**Supplementary Figure S1**

(a) GPx1, LC3 and β-actin expression levels were measured by immunoblot analysis in PDA cells following the silencing of GPx1 in glucose-free medium and were compared with those in the control cells. (b) Expression levels of LC3, SQSTM1 and β-actin in GPx1-silenced cells in in glucose-free medium were examined by western blot analysis following treatment with CQ (10 μM) for 24 h. (c) GPx1-silenced cells in glucose-free medium were treated with Rap (100 nM) for 24 h, and the indicated protein levels were then analyzed by western blotting. (d) MiaPaCa-2 cells in glucose-free medium were transfected with GFP-mRFP-LC3B and subsequently treated with CQ (10 μM) or Rap (100 nM) for 24 h and were then observed using a confocal microscope for the change in both green and red fluorescence (Scale bar: 20 μm). The number of acidified autophagosomes (GFP^-^RFP^+^) versus the number of neutral autophagosomes (GFP^+^RFP^+^) per cell in each condition are quantified in (e) (**P* < 0.05, ***P* < 0.01).

**Supplementary Figure S2**

(a-b) GPx1 and LC3 expression levels were negatively correlated in the PDAC tissue samples (n = 40, Spearman r = -0.3647, **P* < 0.05).

**Supplementary Figure S3**

(a-b) ECAR and OCR analyses were performed in PANC-1 cells with GPx1 overexpression cultured in glucose-free medium. (c) ATP production was measured in PNNC-1 cells with GPx1 overexpression cultured in glucose-free medium (n.s., no significance, **P* < 0.05).

**Supplementary Figure S4**

(a-b) The mRNA and protein levels of GLUT1, LDHA, PDK1 and HK2 were further decreased by GPx1 knockdown cultured in glucose-free media (***P* < 0.01).

**Supplementary Figure S5**

These mice were randomly divided into two subgroups formed by SW1990 cells stably silencing GPx1 or empty vector (n = 5/each group). The expression of the enzymes GLUT1, HK2, PKM2, and LDHA decreased in tumor tissue sections from the xenografts formed by GPx1 silencing in SW1990 cells using IHC assay (scale bar, 20 μm).

**Supplementary Figure S6**

MiaPaCa-2 and SW1990 cells were incubated in DMEM or glucose-free medium for 24 h. Cell lysates were immunoblotted for SOD1, PON1, CAT, GPx1 and β-actin.
