# Supplementary figures and images for: GPx1 is involved in the induction of protective autophagy in pancreatic cancer cells in response to glucose deprivation
Source: Cell Death Dis. 2018 Dec 11;9(12):1187. doi: 10.1038/s41419-018-1244-z (PMC6290009; doi:10.1038/s41419-018-1244-z)

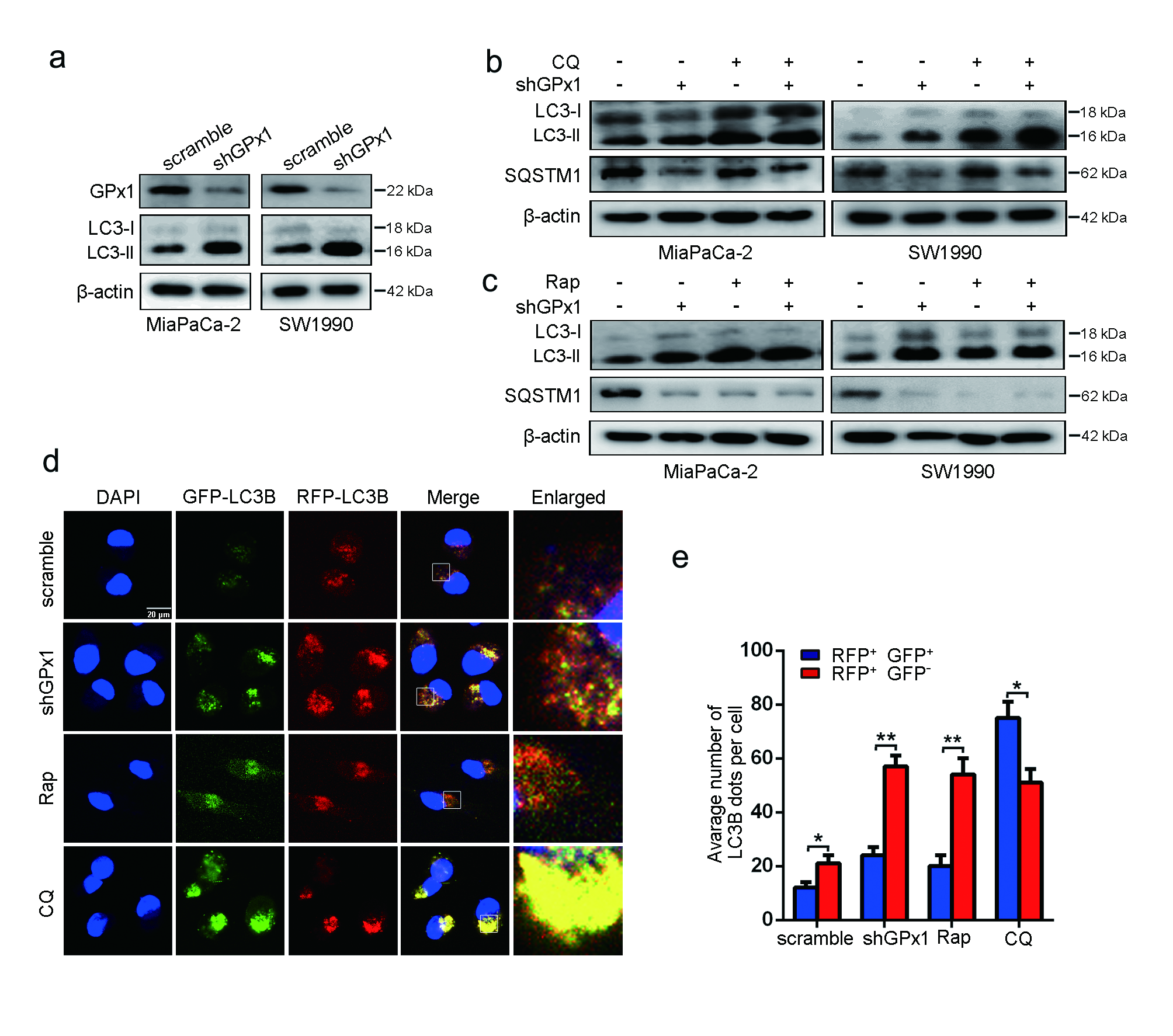

Supplement: Supplementary file 2 — Supplementary Figure 1 [file 41419_2018_1244_MOESM2_ESM.tif]

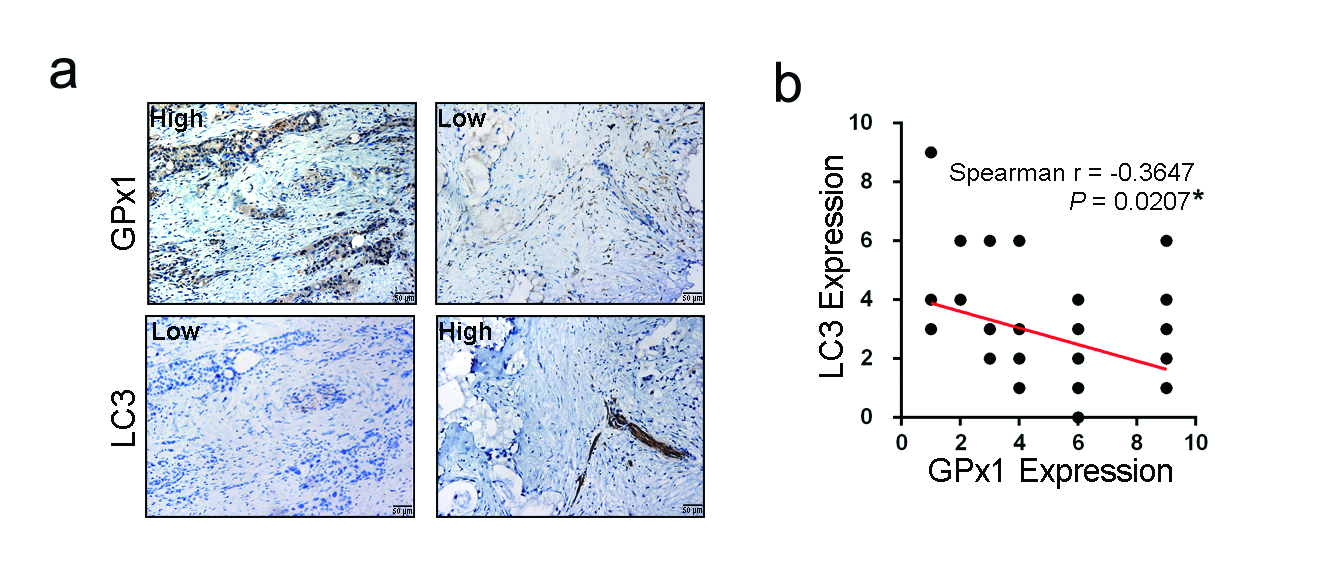

Supplement: Supplementary file 3 — Supplementary Figure 2 [file 41419_2018_1244_MOESM3_ESM.tif]

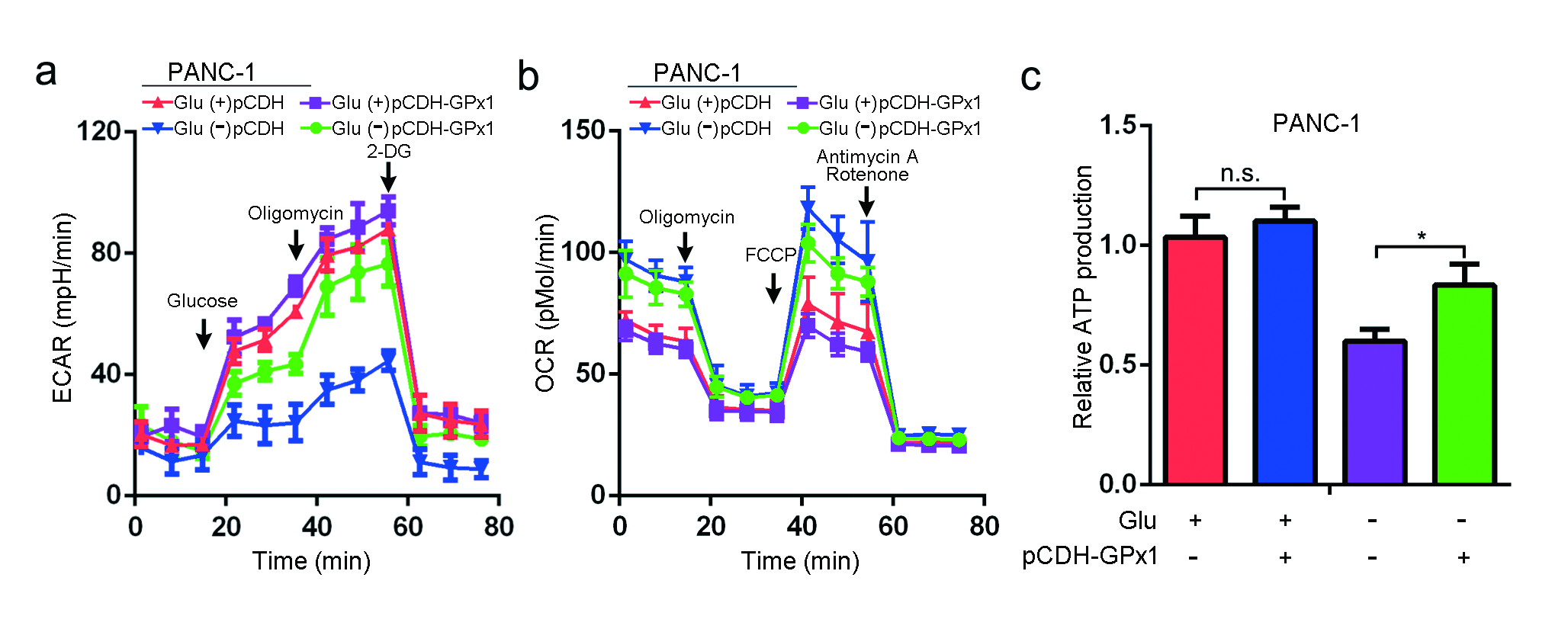

Supplement: Supplementary file 4 — Supplementary Figure 3 [file 41419_2018_1244_MOESM4_ESM.tif]

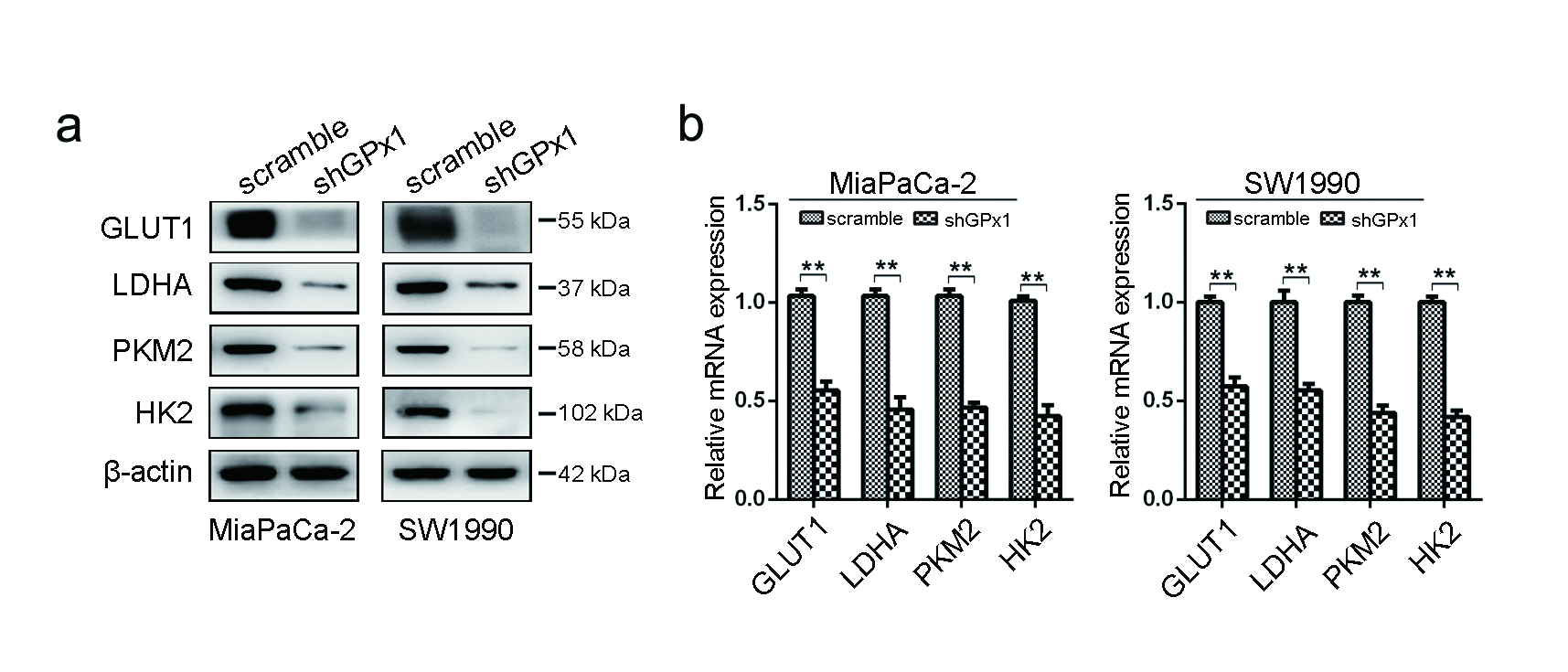

Supplement: Supplementary file 5 — Supplementary Figure 4 [file 41419_2018_1244_MOESM5_ESM.tif]

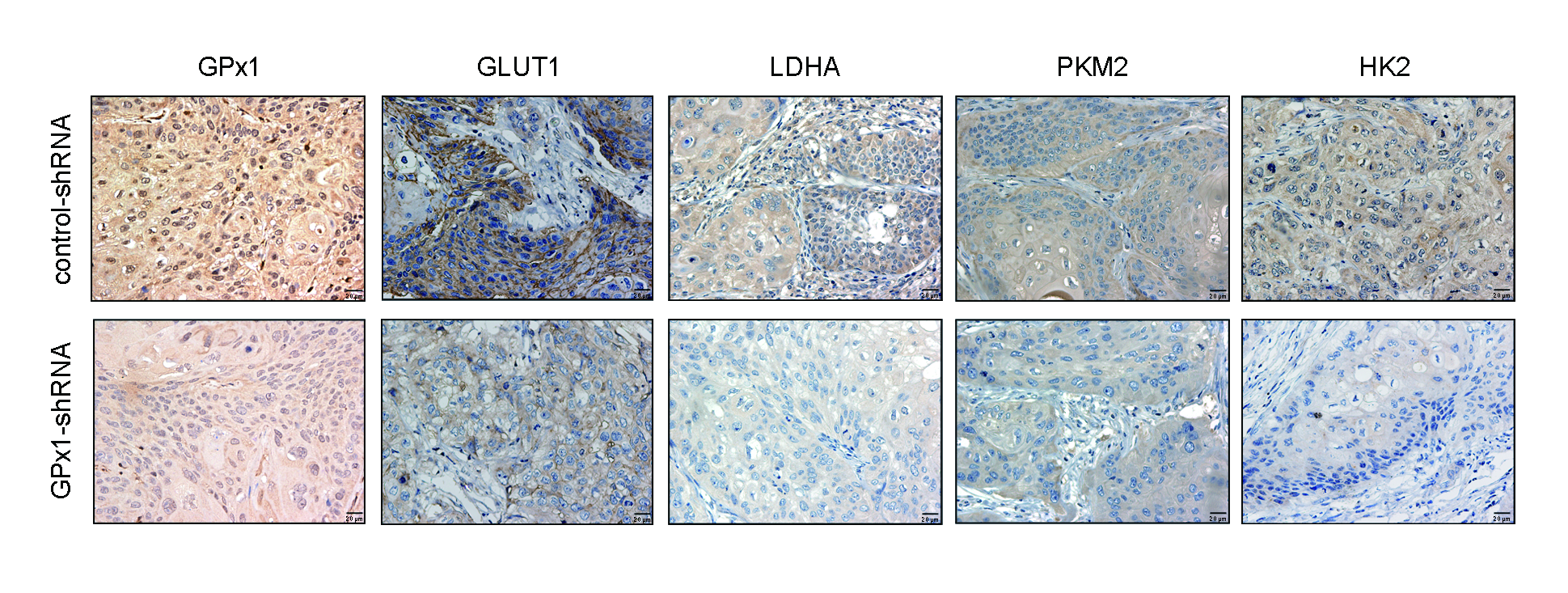

Supplement: Supplementary file 6 — Supplementary Figure 5 [file 41419_2018_1244_MOESM6_ESM.tif]

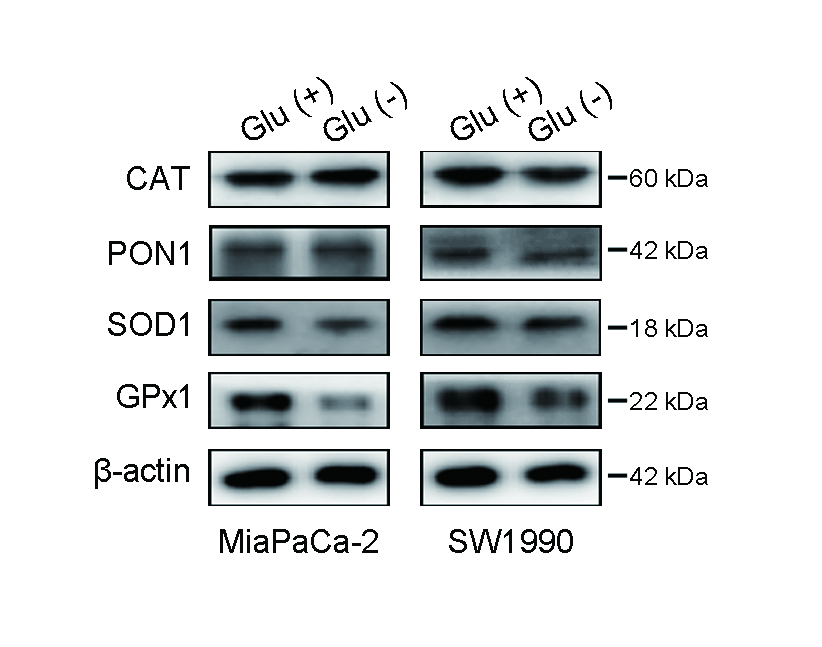

Supplement: Supplementary file 7 — Supplementary Figure 6 [file 41419_2018_1244_MOESM7_ESM.tif]
