# Supplementary material for: GPx1 is involved in the induction of protective autophagy in pancreatic cancer cells in response to glucose deprivation
Source: Cell Death Dis. 2018 Dec 11;9(12):1187. doi: 10.1038/s41419-018-1244-z (PMC6290009; doi:10.1038/s41419-018-1244-z)
Supplement: Supplementary file 8 — Supplementary Table 1 [file 41419_2018_1244_MOESM8_ESM.docx]

**Supplementary Table 1. Primer sequences used in the study.**

| **Gene Name** | **Direction** | **Primer Sequence (5'-3')** |
| --- | --- | --- |
| GLUT1 | Forward | CTTTGTGGCCTTCTTTGAAGT |
|  | Reverse | CCACACAGTTGCTCCACAT |
| HK2 | Forward | GATTGTCCGTAACATTCTCATCGA |
|  | Reverse | TGTCTTGAGCCGCTCTGAGAT |
| PKM2 | Forward | CAAAGGACCTCAGCAGCCATGTC |
|  | Reverse | GGGAAGCTGGGCCAATGGTACAGA |
| LDHA | Forward | TGGAGATTCCAGTGTGCCTGTATGG |
|  | Reverse | CACCTCATAAGCACTCTCAACCACC |
| GPx1 | Forward | TGCTCGGCTTCCCGTGCAACCAGT |
|  | Reverse | GGTGATGAGCTTGGGGTCGGTCAT |
| β-actin | Forward | CTACGTCGCCCTGGACTTCGAGC |
|  | Reverse | GATGGAGCCGCCGATCCACACGG |
